# YOLOv5-AC: Attention Mechanism-Based Lightweight YOLOv5 for Track Pedestrian Detection

**DOI:** 10.3390/s22155903

**Published:** 2022-08-07

**Authors:** Haohui Lv, Hanbing Yan, Keyang Liu, Zhenwu Zhou, Junjie Jing

**Affiliations:** School of Automation, Chengdu University of Information Technology, Chengdu 610225, China

**Keywords:** pedestrian detection, deep learning, model pruning, context extraction module, attention module, DIoU_NMS

## Abstract

In response to the dangerous behavior of pedestrians roaming freely on unsupervised train tracks, the real-time detection of pedestrians is urgently required to ensure the safety of trains and people. Aiming to improve the low accuracy of railway pedestrian detection, the high missed-detection rate of target pedestrians, and the poor retention of non-redundant boxes, YOLOv5 is adopted as the baseline to improve the effectiveness of pedestrian detection. First of all, L1 regularization is deployed before the BN layer, and the layers with smaller influence factors are removed through sparse training to achieve the effect of model pruning. In the next moment, the context extraction module is applied to the feature extraction network, and the input features are fully extracted using receptive fields of different sizes. In addition, both the context attention module CxAM and the content attention module CnAM are added to the FPN part to correct the target position deviation in the process of feature extraction so that the accuracy of detection can be improved. What is more, DIoU_NMS is employed to replace NMS as the prediction frame screening algorithm to improve the problem of detection target loss in the case of high target coincidence. Experimental results show that compared with YOLOv5, the AP of our YOLOv5-AC model for pedestrians is 95.14%, the recall is 94.22%, and the counting frame rate is 63.1 FPS. Among them, AP and recall increased by 3.78% and 3.92%, respectively, while the detection speed increased by 57.8%. The experimental results verify that our YOLOv5-AC is an effective and accurate method for pedestrian detection in railways.

## 1. Introduction

As rail transportation plays an increasingly important role in China, the safety of rail transit operations has also attracted more and more attention. However, in some remote areas, the train track crosses the highway and pedestrian passage. In particular, pedestrians still stay on the track when the train is about to arrive, which will bring huge potential safety hazards, and accidents occur frequently. These pedestrians usually move fast and irregularly on the railway track, while the target is very small and has a high degree of coincidence of body positions within the visual range of the machine’s vision. In addition, complex and uncertain environmental factors such as trees, weeds, and telephone poles around the railway track have caused huge obstacles to pedestrian detection. It is of great significance to carry out research on pedestrian detection and abnormal state monitoring at railway stations to ensure the safety of pedestrians.

Traditional machine learning target detection algorithms, such as the Viola–Jones Detector, generally use the sliding window method to extract candidate frames. They first extract and learn low and intermediate features in candidate frames, and then use classifiers to identify and select objects, which makes it difficult to solve the problems caused by fast movement, small targets, high randomness of appearance, and the high degree of coincidence of body positions. In order to better deal with these difficulties, we propose a detection algorithm based on deep learning, which can help us to obtain a better detection effect by learning the higher-level features of the object through Convolutional Neural Networks (CNNs) [1]. The deep learning target detection algorithm has been in development since R. Girshick et al. proposed Region-CNN (RCNN) [2] in 2014. Since then, Fast R-CNN [3], Faster R-CNN [4], Spatial Pyramid Pooling (SPP) [5], two-stage detectors, You Only Look Once (YOLO) [6,7,8,9], Single Shot MultiBox Detector (SSD) [10], and other single-stage detectors have emerged. The two-stage detector uses a convolutional neural network to extract the features of the markers, and then uses Region Proposal Net (RPN) to recommend candidate boxes, which returns the candidate boxes to the predicted position through a gradient descent at the end. Conversely, the single-stage detector directly performs the regression of the bounding box after extracting the features by ignoring the RPN. The two-stage detector uses two different networks to classify and locate objects, so the detection accuracy is at a high level while the speed is very slow, requiring at least 100 ms to detect an image, such as the Faster RCNN. The single-stage detector uses only one network to perform classification and positioning at the same time, so detection speed is guaranteed. The detection speed of YOLOv1 can reach 45–120 fps, which can process video or camera images in real-time, requiring less equipment and achieving better performance in field deployment.

With the development of transportation, pedestrian detection has gradually become a hot spot in the field of target table detection, where many experts and scholars have put forward their views and opinions. Jin, Xianjian et al. proposed a pedestrian detection algorithm based on YOLOv5 in an autonomous driving environment [11]; Gai Y et al. proposed a method of pedestrian detection + tracking + counting based on YOLOv5 with Deepsort [12]; Sukar et al. proposed an improved YOLOv5 algorithm for real-time pedestrian detection [13]. Zhi Xu et al. proposed a method of CAP-YOLO based on channel attention for Coal Mine Real-Time Intelligent Monitoring [14]. Masoomeh Shireen Ansarnia et al. proposed a deep learning algorithm for contextual detection in orthophotography [15]. Kamil Roszyk et al. adopted a method for low-latency multispectral pedestrian detection in autonomous driving by YOLOv4 [16]. Luying Que et al. proposed a lightweight pedestrian detection engine of a two-stage low-complexity detection network and adaptive region focusing technique [17]. Yang Liu et al. used a thermal infrared vehicle and pedestrian detection method in complex scenes [18]. Jingwei Cao et al. proposed a pedestrian detection algorithm for intelligent vehicles in complex scenarios [19]. Isamu Kamoto et al. used a deep learning method to predict crowd behavior based on LSTM [20]. Gopal, D.G. et al. proposed a method of selfish node detection based on evidence by trust authority and selfish replica allocation in DANET [21]. Jerlin, M.A. et al. created a smart parking system based on IoT [22]. Nagarajan, S.M. et al. applied an intelligent anomaly detection framework to cyber physical systems [23]. Selvaraj, A. et al. put forward a swarm intelligence approach of optimal virtual machine selection for anomaly detection [24]. Nagarajan, S.M. et al. put forward an effective task scheduling algorithm with deep learning for IoHT in sustainable smart cities [25]. The above algorithms have put forward corresponding practical innovations in pedestrian detection and processing, but few achievements have been made in railway pedestrian detection, which is one of the most high-risk scenarios. This paper aims to carry on the corresponding research and experiments for this scene.

Aimed at the problem of the low detection accuracy caused by the rapid movement of the target or the prediction frame completely deviating from the target, as well as the missed detection of the target caused by the high coincidence of body positions, an improved target detection algorithm based on YOLOv5s is proposed.

(1) L1 [26] regularization is added to constrain the scaling factor of the BN [27] layer to make the activation coefficients sparse. Next, the modified model is sparsely trained to cut out the sparse layers. We end up with a very compact model with repeated cutting.
(2) In Backbone, the CEM module is introduced to fully extract the features of different scales. The CxAM module is introduced to extract context semantic information to improve recognition accuracy. The CnAM module is introduced to correct the position of F5 layer features and improve the accuracy of target box regression.
(3) DIoU_NMS is used instead of NMS to filter prediction boxes to avoid eliminating different target prediction boxes with high consistency.
(4) We collected a certain number of datasets along with a certain number of relevant public datasets to provide data support for the verification of the actual effect of the improved model.
(5) According to the direction of improvement, a number of related ablation experiments were designed to verify the validity of each contribution.

## 2. Related Works

### 2.1. YOLOv5 Network Structure

The YOLO series of algorithms, from YOLOv1 to YOLOv5 [28], has been the hottest algorithm in the field of target detection due to its fast and efficient performance. The latest generation of YOLOv5’s weight files is only 28 MB, which is ideal as an initial model. Therefore, YOLOv5 is selected as the experimental object for algorithm improvement in this experiment.

**Figure 1:**
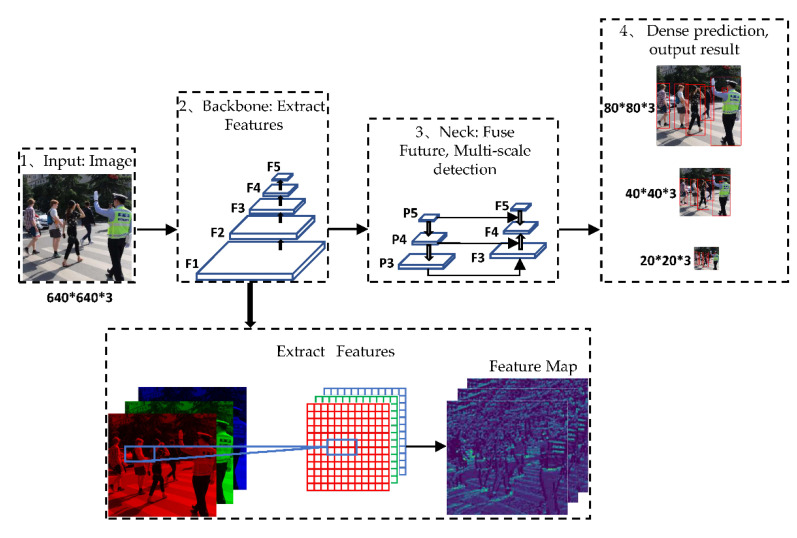
YOLOv5 algorithm structure diagram.

The network structure of YOLOv5 generally follows the previous series. The feature extraction network of the backbone adopts CSPDarknet [29]. The input newly adds the focus structure, slices the input image, reduces the size, and increases the depth, which can improve the speed of feature extraction. At the same time, the CSP2 structure is deployed to the neck part to enhance the ability of network feature fusion. The optimization function adopts Adam [30] and SGD [31]. Focus and conv are the structures that mainly contain the convolution kernel and residual components. As a consequence, the network depth can be changed by controlling the number of residual components in Conv, while the network width can be adjusted by gaining command of the number of convolution kernels in Focus and Conv. Therefore, YOLOv5 has launched four models ranging from small to large by regulating the parameters: YOLOv5s, YOLOv5m, YOLOv5l, and YOLOv5x. The YOLOv5 network structure is shown in Figure 1.

#### 2.1.1. Input

The input layer uses conventional enhancement methods such as rotation, flipping, and blurring, as well as advanced enhancement methods such as Mosaic, and added settings such as adaptive anchor and adaptive image scaling. The dataset images used in the experiment have different lengths, widths, and proportions. They are scaled to the same size of 640 × 640 to reduce training parameters and speed up training and inference.

#### 2.1.2. Backbone

**Figure 2:**
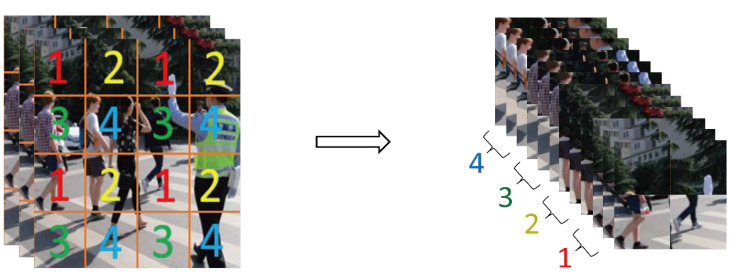
Focus structure diagram.

YOLOv5 uses CSPDarknet as the Backbone for feature extraction, which includes structures such as Focus, CSP, and SPP. Focus is a slicing operation on the Feature map, integrating the information of width w and height h into the c dimension. Specifically, it splices and stacks units 2 pixels apart on a channel, reducing the width and height by 2 times and increasing the number of channels by 4 times, which can reduce Floating Point Operations Per Second (FLOPS) and improve the inference speed. Its structure is shown in Figure 2.

**Figure 3:**
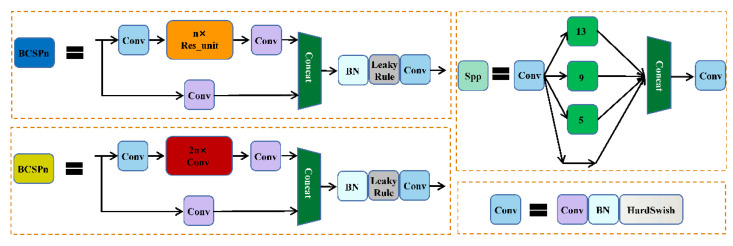
BCSP and SPP structure diagram.

CSP contains two structures, including residual (blue) and excluding residual (yellow). CSPNet solves the problem of network optimization for gradient information repetitions in other large-scale convolutional neural network framework Backbones and integrates gradient changes into feature maps from stem to stern so that the parameter quantity and FLOPS value of the model are decreased, which not only ensures the inference speed and accuracy, but also reduces the model size. Spatial Pyramid Pooling (SPP) is used to pool feature maps at various scales, and concatenate the different results obtained in the channel dimension to increase the receptive field and improve the alignment of Anchor and Feature maps. The structures of CSP and SPP are shown in Figure 3.

#### 2.1.3. Neck

The Neck of YOLOv5 adopts the Feature Pyramid Networks (FPN) [32] structure, performs continuous downsampling to extract features from bottom to top, which can obtain a feature map with decreasing size and pixel value to form a feature pyramid, performs continuous upsampling from top to bottom to restore the feature map size, and fuses with the feature map of the same size on the left to output a multi-scale feature map. FPN has the ability to perform multi-scale result prediction, which can enhance the recognition ability of the model, as well as perform better detection of the same object of different sizes.

#### 2.1.4. Output

YOLOv5 uses NMS as the screening criteria for predicting box regression and takes the overlap area between the predicted box and the real box, the aspect ratio, and the center point distance as the judgment basis. The NMS algorithm is used as the prediction box filtering algorithm to remove the redundant boxes of the same detection target. The final output is 80 × 80, 40 × 40, and 20 × 20 detection results.

## 3. Method

### 3.1. Data Augmentation

The most important part of the data augmentation used by YOLOv5 is the mosaic, which is improved from cutmix. Cutmix is used to scale and crop two pictures and stitch them together according to the pixel ratio.

Mosaic can greatly improve the problem of inaccurate small-target detection by stitching four pictures using the methods of random scaling, random cropping, and random arrangement.

### 3.2. Adaptive Image Scaling

**Figure 4:**
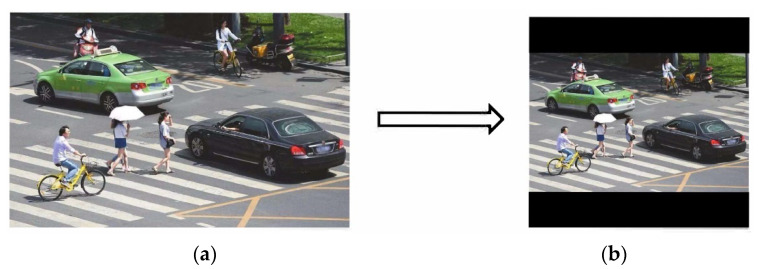
Conventional scaling and filling method. (**a**) Original image: 800 × 600; (**b**) scaled image: 416 × 416.

The length and width of the input images are different when performing target detection, which will affect the efficiency of network computing. Therefore, they are usually scaled to the same standard size and then sent to the detection network. The commonly used input sizes of the YOLO algorithm are 416 × 416 and 608 × 608. Image transformation with a size of 800 × 600 is shown in Figure 4.

In actual projects, many images have different aspect ratios. The black borders at both ends are padded in different sizes after scaling and filling, where there will be information redundancy if there is too much padding, which will affect the inference speed. In YOLOv5, the author proposes a nice trick to help with training by modifying the letterbox function to add minimal black borders to the original image, which can be divided into the following steps:

(1) Calculate the scaling ratio.
(2) Calculate the scaled size.
(3) Calculate the black border fill value.

**Figure 5:**
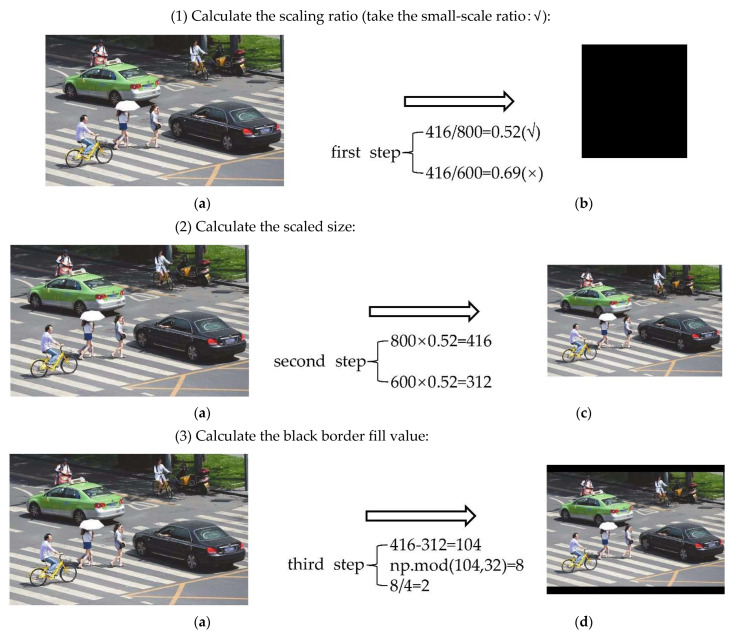
Improved image scaling method. (**a**) Weight × height: 800 × 600; (**b**) weight × height: 416 × 416; (**c**) weight × height: 416 × 312; (**d**) weight × height: 416 × 320 (312 + 4 + 4).

The scaling process is shown in Figure 5.

### 3.3. Loss Function

The loss function is an important indicator for evaluating regression and classification problems. The loss function of YOLOv5 includes Classification Loss, Localization Loss, and Confidence Loss. Classification loss and confidence loss use the binary cross entropy function, its expression is seen in Formula (1):

(1) $$loss=-\frac{1}{N}\sum_{i=1}^{N}y_{i}\cdot \log\left(p\left(y_{i}\right)+\left(1-y_{i}\right)\cdot \log\left(1-p\left(y_{i}\right)\right)\right)$$

where $y_{i}$ is the binary label 0 or 1 and $p\left(y_{i}\right)$ is the probability of the label. The binary cross entropy function is used to judge the quality of the prediction results of the binary classification model. If the predicted label probability is close to 1, then the loss function is close to 0. The target box regression loss uses the CIoU_Loss [33] (Complete Intersection over Union) function as the evaluation index, and its expression is seen in Formula (2),

(2) $$l_{CIoU}=1-IoU+R_{CIoU}$$

where IoU is the area intersection ratio of Ground True box A and Bounding box B, and $R_{CIoU}$ is the penalty factor, which acts as a block of variables that distinguishes different regression loss functions. With different *IoU*_Loss, the form of the penalty function appears different,

(3) $$IoU=\frac{A\cap B}{A\cup B}$$

$R_{CIoU}$ is defined as

(4) $$R_{CIoU}=\frac{\rho^{2}\left(b,b^{gt}\right)}{C^{2}}+\alpha v$$

where b and $b^{gt}$ denote the central points of B and $B^{gt}$, ρ is the Euclidean distance, and C is the diagonal length of the smallest enclosing box covering the two boxes. α is the trade-off parameter and v is the relationship factor between the width and height of the prediction frame and the coordinates of the center point

(5) $$\alpha =\frac{v}{(1-IoU)+v}$$

Then, the relationship factor v is defined as

(6) $$v=\frac{4}{\pi^{2}}{\left(\arctan\frac{w^{gt}}{h^{gt}}-\arctan\frac{w}{h}\right)}^{2}$$

where w is the width of the prediction box, h is the height of the prediction box, $w^{gt}$ is the width of the ground truth box, and $h^{gt}$ is the height of the ground truth box.

### 3.4. Sparse Training and Model Pruning

YOLOv5 is already a cracking lightweight detection network where the trained weight model generally does not exceed 30 MB, which is still too large for some embedded devices. If we simply choose to reduce the size of the network input, such as 640 to 320, as the size of the model is reduced accordingly the detection effect will also have a greater loss at the same time. Therefore, according to a method of network slimming proposed by Zhuang Liu et al. [34], we add L1 regularization parameters to the model to constrain the scaling factor of the BN layer, which can cause the coefficients close to 0 to become smaller. These pairs of parameter layers with little influence on forward propagation are eliminated through sparse training. We can obtain a very compact and efficient network model by repeating the above operations.

The loss function expression with L1 regularization is seen in Formula (7)

(7) $$L=\sum_{\left(x,y\right)}l\left(f\left(x,W\right),y\right)+\lambda \sum_{\gamma }g\left(\gamma \right)$$

Among them, the former term is the usual network loss function and the latter term is the regularization of the scale factor where x, y denote the train input and target, W denotes the trainable weights, g is a sparsity-induced penalty on the scaling factors, γ is the scale factor, and λ is the penalty sparse parameter that determines the size of the penalty term. The L1 regular expression is seen in Formula (8)

(8) g(s)=|s|

BN layer parameters are calculated as follows:

(9) $$\hat{x}=\frac{z_{in}-\mu_{Β}}{\sqrt{\sigma_{Β}^{2}+\epsilon }}$$

(10) $$z_{out}=\gamma \hat{z}+\beta$$

where $\mu_{B}$ and $\sigma_{B}$ are the mean and variance of the activations, while γ and β are the trainable affine transformation parameters. $z_{in}$ and $z_{out}$ are the input and output of a BN layer. We choose to use the BN layer γ as the scale factor for sparsity clipping directly.

**Figure 6:**
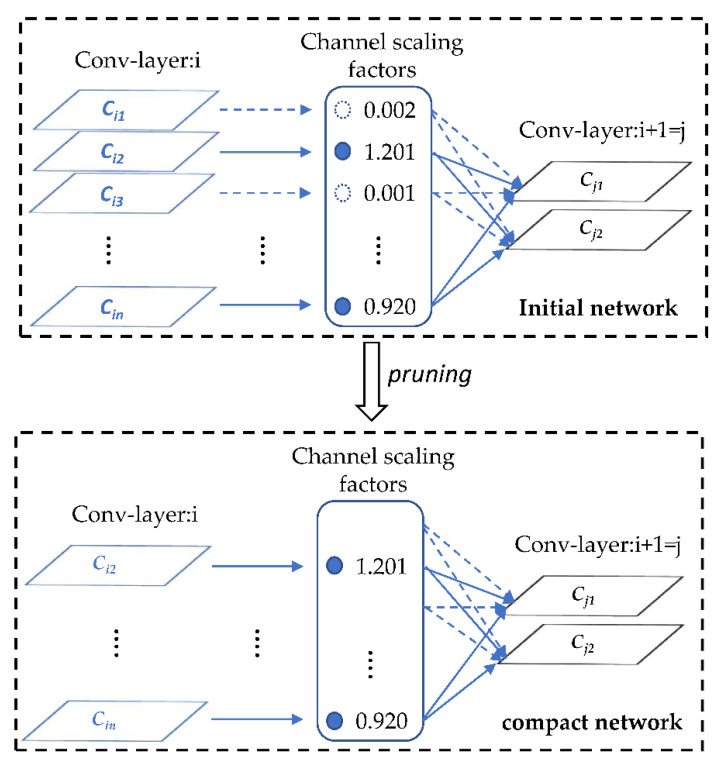
Principle of pruning.

The principle of YOLOv5 network channel clipping is shown in the Figure 6.

**Figure 7:**
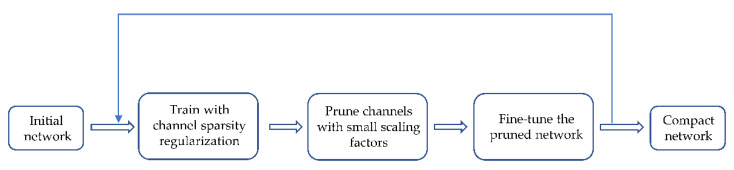
Process of pruning.

Above all, we append L1 regularization to the model to perform corresponding sparse training. Then, channel pruning is performed on the trained model. Ultimately, the training hyperparameters are fine-tuned to ensure the model inference results are optimal. The algorithm implementation process is shown in Figure 7.

### 3.5. Improved AC_FPN

The higher the resolution of the input image in the training network, the more feature information needs to be extracted, and the more requirements are put forward for the receptive field of the convolution layer. The general convolutional neural network uses multiple convolution and pooling operations. In order to improve the receptive field, the size of the feature map should be reduced. However, when up-sampling the feature map to restore its original size, a great deal of feature information is lost, which causes bias in the final classification result and prediction regression. In order to avoid this situation, this paper improves the Feature Pyramid Networks (FPN) structure and sends the feature map of the highest F5 layer of the feature pyramid to the Context Extraction Module (CEM) for multi-scale hole convolution so the features of different scales will be fully extracted. Then, the extracted feature information is sent to Context Attention Modules (CxAM) for contextual semantic information extraction to determine the target more accurately. At the same time, it is sent to Content Attention Modules (CnAM) to compare the features of the F and F5 layers, correcting the position shift that occurs in the feature extraction process, performing more accurate frame selection on the target. Finally, the processed feature map and the feature map of the deconvolution layer are multi-scale fusion, which outputs the prediction result after the algorithm process. FPN is composed of an upward convolution pyramid and a downward deconvolution pyramid. The feature maps of the same size are fused at multiple scales through horizontal connections in the middle. FPN can enhance the effect of feature extraction compared with traditional convolutional networks.

#### 3.5.1. CEM

**Figure 8:**
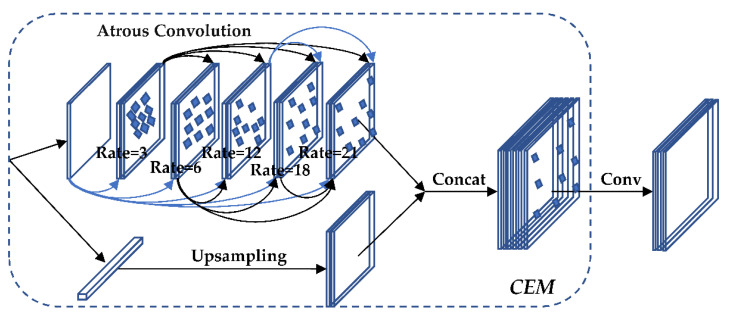
CEM structure diagram.

The CEM module is a feature pyramid structure composed of separable convolutional layers with different void rates. The void rate gradually increases from 3 → 6 → 12 → 24 where the layers are connected by a multi-path, and the different receptive fields of each layer are used to fully extract the features so as to realize the diversity of features in scale. The structure of the CEM is shown in Figure 8.

#### 3.5.2. CxAM

Since there is no screening mechanism, many useless features will be sent to the end of the network for prediction after features are fully extracted by CEM, which will affect the accuracy of the output results. Therefore, the attention module (AM) is set to eliminate useless features. AM contains two submodules, CxAM and CnAM.

**Figure 9:**
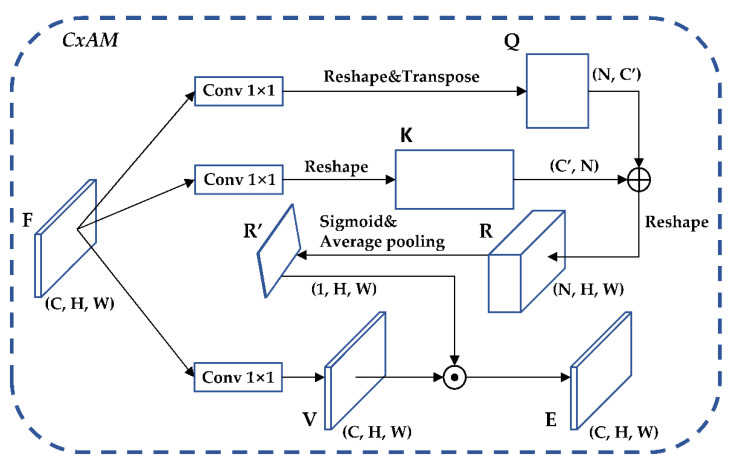
CxAM structure diagram.

CxAM: Contextual Attention Module, which inputs the same feature of different scales extracted by CEM, extracts the semantic relationship between different feature sub-regions, and uses the feature map to generate the attention matrix. As a result, the output feature map will contain clear semantic information. The structure of the CxAM is shown in Figure 9.

#### 3.5.3. CnAM

**Figure 10:**
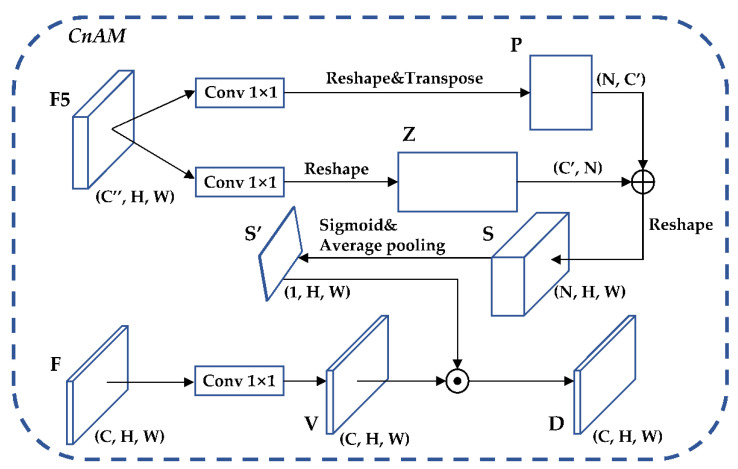
CnAM structure diagram.

CnAM: Content Attention Module, which uses the F-layer feature map to calibrate the position offset that occurs in the feature extraction process to obtain more accurate box selection positioning when it is used for target detection. The structure of the CnAM is shown in Figure 10.

#### 3.5.4. AC_FPN Structure

**Figure 11:**
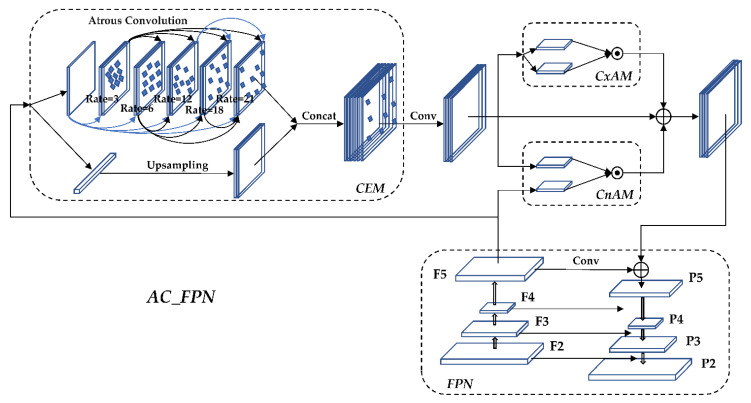
AC_FPN structure diagram.

The output is sent to the deconvolution layer of the FPN network for feature fusion after the feature maps are processed by the above three modules. The improved AC_FPN [35] structure is shown in Figure 11.

### 3.6. Improved NMS

Non-Maximum Suppression (NMS) needs to be performed for the screening of many target boxes in the post-processing process of target detection. YOLOv5 adopts the traditional NMS [36] method. The occluded target selection box is usually removed when facing two different targets with a high degree of coincidence by using this method. For an environment with a large number of targets, where there will be many targets with a high degree of coincidence, the occlusion target candidate boxes that are obscured will be removed as redundant information by NMS, which is not suitable for models that want to detect accurately. In this paper, DIoU_NMS is used to replace the NMS. DIoU_NMS introduces the parameter *β* of the center point distance between the two boxes. When *β* → ∞, DIoU_NMS degenerates into traditional NMS. Otherwise, as long as the center points of the two frames do not coincide perfectly when *β* → 0, they will be retained by DIoU_NMS. As a consequence, the value of *β* can be adjusted to 0 → ∞ according to the actual situation to achieve the best effect to restrain redundant boxes. Its classification score update formula is defined as Formula (11):

(11) $$s_{i}=\{\begin{array}{l} s_{i},IoU-R_{DIoU}\left(M,B_{i}\right)<\epsilon \\ 0,IoU-R_{DIoU}\left(M,B_{i}\right)\geq \epsilon \end{array},$$

where $s_{i}$ is the classification score and ϵ is the NMS threshold, $R_{DIoU}\left(M,B_{i}\right)$ is the penalty item, M is the predicted box with the highest score, and $B_{i}$ is the other box.

Among them is the highest-class score of the prediction box, which is the threshold of the distance between the center points of the two prediction boxes. DIoU_NMS suggests that the two boxes with farther center points may be located on different objects, thus it should not be deleted, which is the biggest difference between DIoU_NMS and NMS.

### 3.7. Improved YOLOv5-AC Network Structure

**Figure 12:**
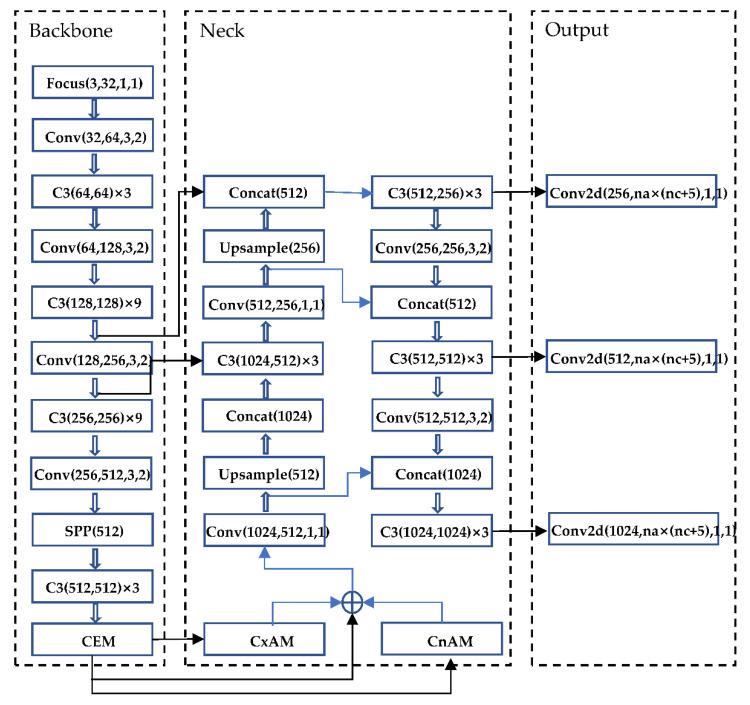
YOLOv5-AC structure diagram.

The features are further extracted by adding a context extraction model (CEM) in Backbone. CxAM is added to extract the context semantics. CnAM is applied to correct the feature positions of the F and F5 layers. Post-processing uses DIoU_NMS to replace NMS. The improved YOLOv5-AC structure is shown in Figure 12.

## 4. Experiment

### 4.1. Datasets

The training data use the collected images and videos of a certain abandoned railroad track near Chengdu Shenxianshu subway station and surrounding pedestrians, combined with screenshots, frame extraction, and other methods to select 5000 of them as the benchmark dataset.

### 4.2. Data Annotations

**Figure 13:**
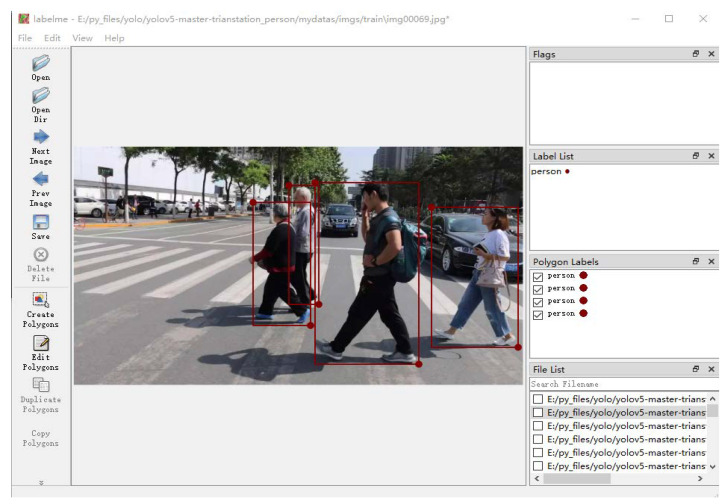
Labelme annotation tool.

We used the labelme tool to annotate the image, which converts the resulting json annotation file to a txt file. The information contained in the txt file includes the type and number of the labeling target, the normalized width and height of the labeling frame, and the coordinates of the center point. The effect of labeling is shown in Figure 13.

**Figure 14:**
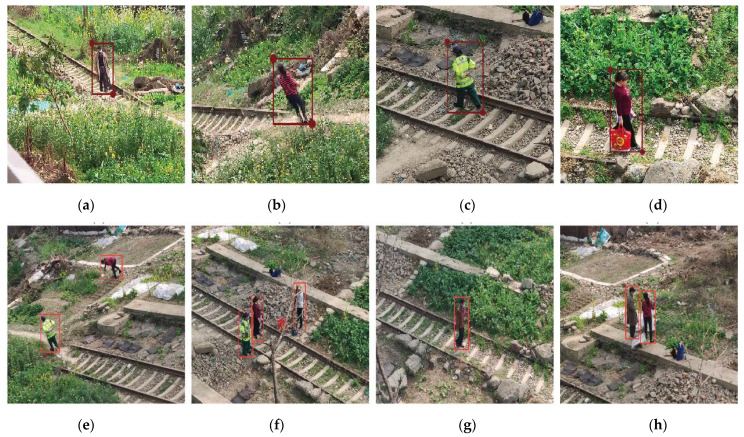
Self-made dataset annotation example. (**a**) self-made dataset 1; (**b**) self-made dataset 2; (**c**) self-made dataset 3; (**d**) self-made dataset 4; (**e**) self-made dataset 5; (**f**) self-made dataset 6; (**g**) self-made dataset 7; (**h**) self-made dataset 8.

The part of the self-made pedestrian dataset marked with the labelme tool is shown in Figure 14.

**Figure 15:**
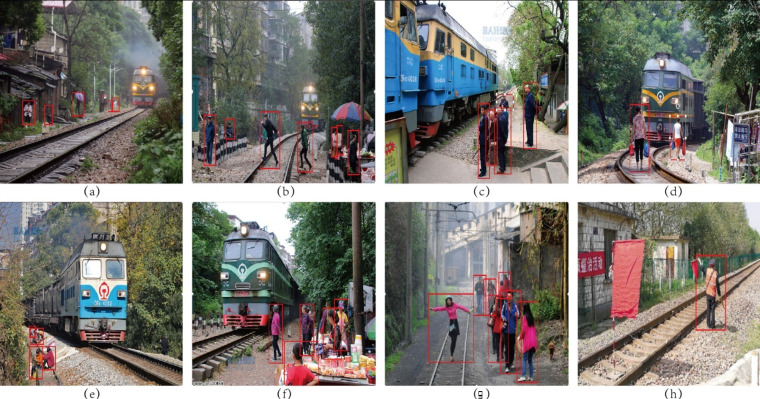
Public dataset annotation example. (**a**) public dataset 1; (**b**) public dataset 2; (**c**) public dataset 3; (**d**) public dataset 4; (**e**) public dataset 5; (**f**) public dataset 6; (**g**) public dataset 7; (**h**) public dataset 8.

The public dataset is labeled as shown in Figure 15.

At the end of the labeling, the corresponding labeling data are obtained, which include the normalized width, normalized height, and normalized center point coordinates x and y of the frame selection target. The information of the self-made data marked in self-made dataset is shown in Table 1 (Note: Classification 0 is a person).

### 4.3. Training Process

This experiment will proceed as shown in Table 2.

This experiment uses a UBUNUTU20.04 system computer, the graphics card RTX3090 (video memory: 24 GB), and the CUDA11.4 computing framework. The specific configuration parameters of the experiment are shown in Table 3.

### 4.4. Training Metrics

*Accuracy* and *Recall* are selected as metrics to compare the quality of the original model and the improved model of the test results. The calculation formulas of *Accuracy* and *Recall* are as follows:

(12) $$Recall=\frac{TP}{TP+TN}$$

(13) $$Accuracy=\frac{TP+TN}{TP+TN+FP+FN}$$

*TP* is the number of people on the track that were correctly detected. *FP* is the number of people on the track that were incorrectly detected as people. *TN* is the number of people on the track that were not detected. *FN* represents no one on the track and no one detected at the same time. The relationship can be intuitively understood through the following confusion matrix Table 4.

## 5. Results

### 5.1. Comparative Experimental Results

**Figure 16:**
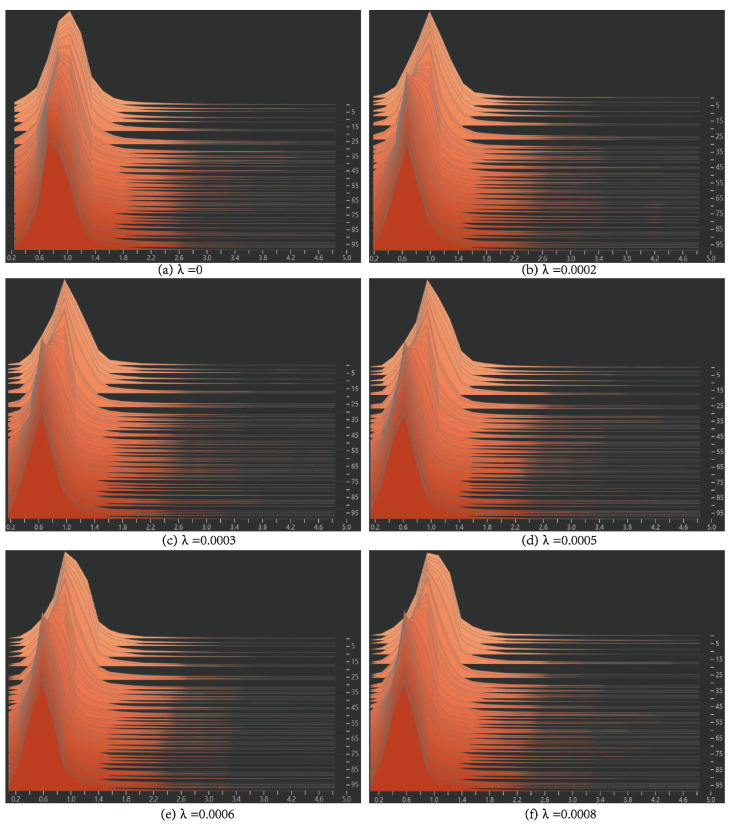
The data distribution of the BN layer when λ changes from 0 to 0.0008.

We observe the parameter distribution of the BN layer by modifying the sparse parameter of the L1 regularity λ from 0 → 0.0008, of which the interval of each change is 0.0001. When λ = 0 for normal training, the distribution curve is roughly the standard state distribution, as shown in Figure 16a below, and there are only a few values near 0 so it is difficult to prune. After adding the penalty term with coefficients, the parameters of the BN layer gradually tend to be near 0 from the normal distribution as the training goes on. At this point, we can select the model to prune. When λ changed from 0 → 0.0008, we conducted training experiments for 100 epochs for each group, and the BN layer parameters change as shown in Figure 16.

λ and the maximum pruning ratio, as well as the corresponding pruned models’ parameters, are shown in Table 5.

From Table 5, we can see that when λ is 0.6 or 0.7, the model can obtain the maximum cropping ratio, in which time the cropped model FLOPS, model size, and other parameters can achieve the best. Therefore, this experiment selects λ = 0.6 as the penalty coefficient of L1 regularity.

After 40% scaling, the total number of network parameters is reduced from 7,063,542 to 3,374,814, while the weight file is reduced from 27.2 MB to 13.1 MB. The details of the network parameters are shown in Table 6.

This experiment uses YOLOv5s as the baseline algorithm, and uses Faster R-CNN, YOLOv4, and other algorithms as comparisons with our improved YOLOv5-AC at the same time. The AP and recall change curves are shown in Figure 16.

**Figure 17:**
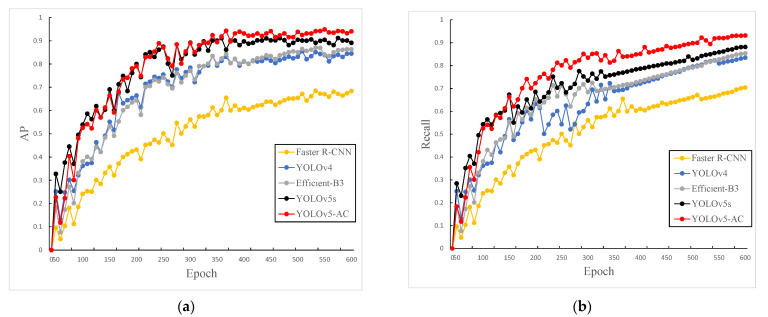
Variation trend of training metrics in the experiment. (**a**) Variation trend of AP. (**b**) Variation trend of Recall.

From Figure 17, it can be seen that the improved YOLOv5-AC algorithm in this experiment is better than the mainstream detection algorithms such as YOLOv5s and Faster R-CNN in terms of AP and Recall.

**Figure 18:**
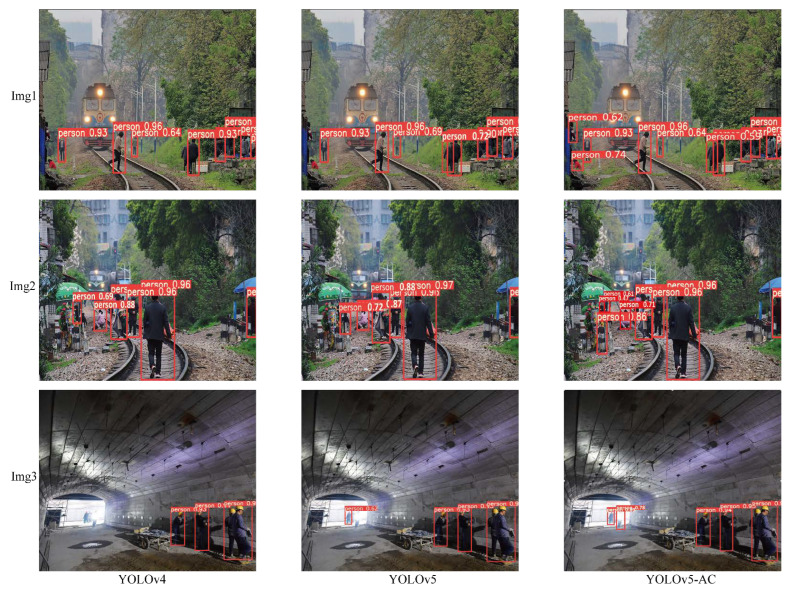
Comparison of training results of YOLOv5-AC and YOLOv4 and YOLOv5.

**Figure 19:**
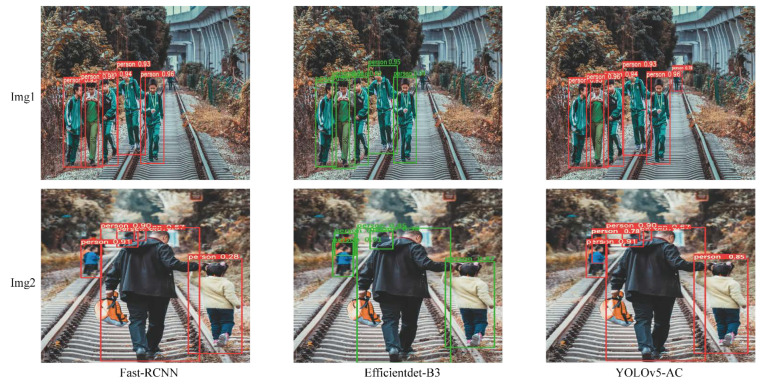
Comparison of training results of YOLOv5-AC and Fast-RCNN and Efficientdet-B3.

The simulation results of pedestrian detection for each algorithm used in the experiment are shown in Figure 18 and Figure 19.

The specific experimental index data are shown in Table 7. As can be seen from Table 7, the AP of improved YOLOv5-AC is 3.78% higher than that of YOLOv5s, and the Recall is 3.82% higher than that of YOLOv5s. At the same time, compared with other mainstream algorithms, such as YOLOv4, Efficientdet, etc., the advantages in parameters, such as the number of parameters, floating-point calculation amount, and FPS, are even more obvious.

From all the above experimental results, the performance of the YOLOv5-AC algorithm is significantly improved compared with the mainstream detection algorithms, including YOLOv5, after pruning the model, improving the attention mechanism, and the non-maximum suppression algorithm.

### 5.2. Ablation Study

This paper uses the channel pruning of the BN layer, AC_FPN module, and DIoU_NMS algorithm to improve the YOLOv5s model. Next, we will verify the effectiveness of each contribution through three groups of ablation studies.

#### 5.2.1. Ablation Study on Network Pruning

In this paper, channel pruning is performed on the YOLOv5-AC model to compress the model size, reduce the total number of parameters, and improve the model inference speed to enhance the actual deployment capability. The following is an ablation experiment on channel pruning to verify the effectiveness. The results are shown in Table 8 (√ means channel pruning, × means no).

**Figure 20:**
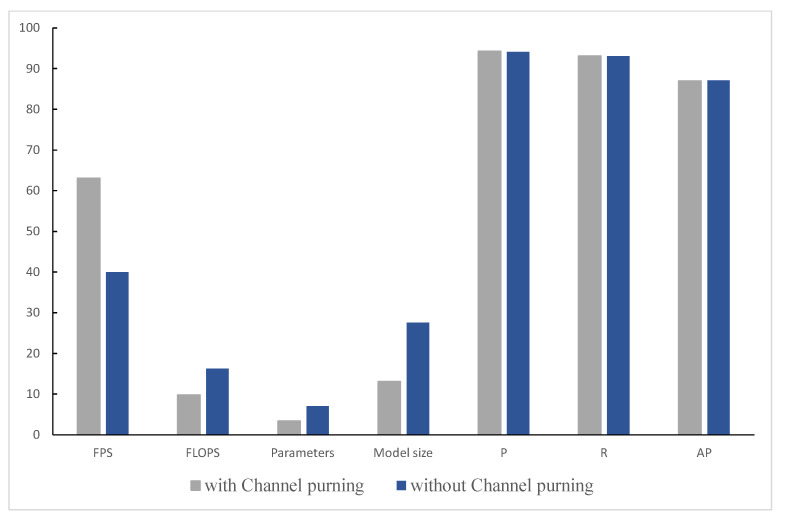
Performance comparison of the model with and without channel pruning.

We can see the comparison of FPS, FLOPS, and other indicators of the model more intuitively with and without channel clipping in Figure 20.

It can be seen from the above table that there is little difference in performance indicators such as P and R with or without pruning of the model. However, after pruning, the FPS of the network is increased by 23.1%, and the FLOPS, Parameters, and Model size are reduced approximately once. The experimental results verify the effectiveness of the channel pruning.

#### 5.2.2. Ablation Study on AC_FPN Model

Table 9 shows the experimental results of the AC_FPN module ablation, where √ indicates that the AC_FPN module is used and × indicates that the original FPN module is used without changing other modules. The use of AC_FPN aims to improve the precision of model detection by adding an attention mechanism. As can be seen from Table 9, AC_FPN is 3.89%, 4.09%, and 3.93% higher than FPN in P, AP, and F1_score. The experimental results verify the effectiveness of the AC_FPN module.

#### 5.2.3. Ablation Study on DIoU_NMS Algorithm

Table 10 shows the results of the DIoU_NMS ablation experiment, where √ indicates that DIoU_NMS is used and × indicates that NMS is used without changing other modules. The addition of DIoU_NMS is used to improve the problem of non-redundant boxes of detection targets with high coincidence being deleted and improve the recall rate. It can be seen from Table 10 that we can improve R and F1_score by 4.17% and 3.55% by using DIoU_NMS. The experimental results verify the effectiveness of the DIoU_NMS algorithm.

### 5.3. Visualization of Heatmaps

**Figure 21:**
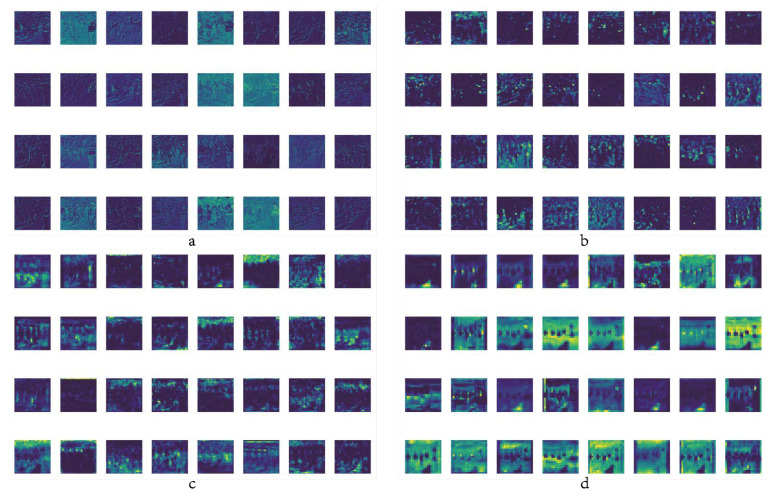
Visualization of feature maps. (**a**) stage1_C3 feature maps; (**b**) stage6_C3 feature maps; (**c**) AC_FPN feature maps; (**d**) output feature maps.

The learning and reasoning process of the neural network is considered to be black-box computing. How the network learns parameters, the respective concerns of each layer of the network, and the interpretability of the deep learning process have always been hot topics in the industry. We will conduct corresponding visualization research on the learning process of the YOLOv5 target detection framework according to the research content of this article in response to this hot topic. Figure 21 is the visualization result of the feature map extracted by the backbone network.

**Figure 22:**
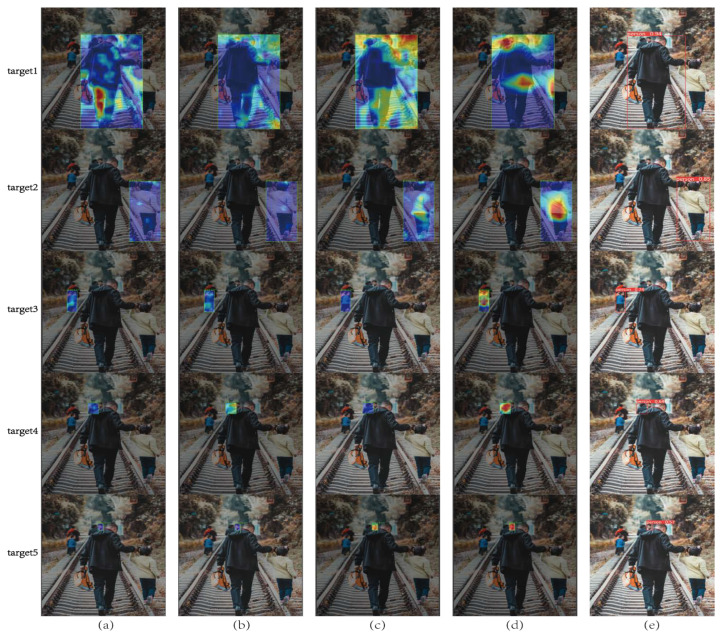
Visualization of heatmaps. (**a**) stage1_C3 heatmaps; (**b**) stage6_C3 heatmaps; (**c**) AC_FPN heatmaps; (**d**) output heatmaps; (**e**) detection results.

As can be seen from Figure 21, with the advancement of the network learning process, the extraction progress of the feature map gradually deepens, the useless features are continuously screened and excluded, and the required main features are obtained in the end. Figure 22 uses the Gradient-weighted Class Activation Mapping (Grad_CAM) [38] heatmap to visualize the response of the feature map to a learning class during the training process, as well as the output decision-making process. Unlike Class Activation Mapping (CAM) [39], which only performs average pooling on the feature map of the last layer, Grad-CAM performs weighted average pooling on all channels. The mapping result of each convolutional layer to the feature map is obtained after relu activation.

In Figure 22, we use heatmaps of four network layers to show the learning decision process of the network. It can be seen that the first few layers of the network feature learning represented by (a) and (b) are loose. However, the heat map gradually becomes closer to the target after adjusting the attention mechanism of (c) AC_FPN, and the heat point obtained by the output layer is almost completely aligned with the target, so the final detection results (e) are accurate.

## 6. Discussion

Compared with YOLOv5s, the improved YOLOv5-AC model has been greatly improved in terms of precision, recall, model size, and FPS. Moreover, the missed detection rate of overlapping targets has been greatly improved, which is a greatly effective algorithm improvement. However, during the experiment, we also found that the algorithm’s ability to recognize small overlapping targets at long distances still needs to be improved. In the future, we will further improve our YOLOv5-AC based on this problem, which is mainly divided into the following two points: First, there are fewer small-target feature areas, so we will perform further data enhancement to improve the sample quality; second, we will use the idea of a sliding window, where the image is divided into n small areas to be detected separately, and the normal image size is concatenated in the end.

Furthermore, we will use the intelligent robot based on jetson nano as the research object. In the meantime, cooperating with the SLAM algorithm and the path planning algorithm to realize pedestrian detection in the process of automatic driving of the robot, we will test the actual effect of our YOLOv5-AC algorithm to help the development and application of pedestrian detection methods in railway traffic.

## 7. Conclusions and Future Works

In view of the accuracy and recall rate of pedestrian detection on train tracks, the corresponding train track datasets were collected, and Labelme was used for manual annotation. We have made improvements to the YOLOv5s deep learning framework. Above all, the L1 regularization function is added to the BN layer, which can remove the network layer with a small impact factor, reducing the size of the model to improve the inference speed. Then, a CEM module is applied to the FPN layer to extract as many features as possible. At the same time, CxAM and CnAM, which are two attention modules, are deployed to filter out the useless features, which can correct the position shift that occurs in the process of feature restoration and improve the detection accuracy. In the end, the DIoU_NMS algorithm is used as the prediction box screening algorithm to reduce the loss rate of non-redundant boxes and improve the recall rate. The final experimental results show that the AP can reach 95.14% and the recall rate can reach 94.22% for the improved YOLOv5-AC algorithm. In addition, the trained weight file is 13.1 MB, and the FPS is 63.1 f/s. YOLOv5-AC shows great improvement in each detection performance compared with YOLOv5s, and the model size and inference speed are better, which facilitate the deployment of actual projects. Our YOLOv5-AC can be used in practical projects related to pedestrian detection on train tracks to improve detection accuracy, reduce missed detection rates, reduce accidents caused by pedestrians randomly crossing the track, and ensure the safety of life and property.

## Figures and Tables

**Table 1:** Information of annotated data.

Picture Number	Classification	Width	Height	Center Point x	Center Point y
(a)	0	0.2202	0.5341	0.1023	0.2346
(b)	0	0.4468	0.6052	0.2012	0.1436
(c)	0	0.4256	0.6214	0.0956	0.1878
(d)	0	0.3218	0.7014	0.1956	0.3125
(e)	0	0.3746	0.8021	0.4126	0.5012
(f)	0	0.3013	0.6589	0.2012	0.6396
(g)	0	0.2017	0.6478	0.6712	0.3125
(h)	0	0.1218	0.2014	0.2313	0.2410

**Table 2:** Procedure of the experiment.

Procedure of the Experiment
Step1: Carrying out pruning experiments to select the L1 parameter that makes the pruning rate, P and R optimal.
Step2: Training YOLOv5s to get training metrics.
Step3: Training YOLOv5-AC to get training metrics.
Step4: Contrasting the results of step2 and step3.
Step5: Comprehensive comparison of mainstream models such as YOLOv5-AC and Faster R-CNN, YOLOv4, Efficientdet-B3 [37].
Step6: A series of ablation trial are designed to verify the validity of every contribution.

**Table 3:** Training parameters.

Training Parameters	Value (Category)
Epoch	600
Batch size	18
Image size	640 × 640
Selected model	YOLOv5s, YOLOv5-AC
Model scaling factor	depth: 0.33 width: 0.50
Total number of parameters	7,063,542
The total number of layers in the model	283

**Table 4:** Confusion matrix.

	Real Situation Somebody	Real Situation Nobody
Predicted somebody	*TP*	*FP*
Predicted nobody	*TN*	*FN*

**Table 5:** The maximum pruning ratio and network parameters when λ changes from 0 to 0.0009.

λ	Max Pruning Ratio (%)	FLOPS(G)	Parameters/106	Model Size (M)
0	10.1	13.2	6.40	24.7
0.0002	24.3	11.6	4.75	18.3
0.0003	33.2	10.6	3.90	15.1
0.0005	37.2	10.1	3.53	13.7
0.0006	39.0	9.7	3.36	13.0
0.0008	36.0	10.0	3.64	13.2

**Table 6:** Pruned network parameters.

Layers	From	Parameters	Module	Arguments
0	−1	3520	Focus	[3, 32, 3]
1	−1	18,560	Conv	[32, 64, 3, 2]
2	−1	18,420	C3	[64, 64, 1]
3	−1	43,492	Conv	[64, 128, 3, 2]
4	−1	142,297	C3	[128, 128, 3]
5	−1	48,506	Conv	[128, 256, 3, 2]
6	−1	559,377	C3	[256, 256, 3]
7	−1	722,356	Conv	[256, 512, 3, 2]
8	−1	162,771	SPP	[512, 512, [5, 9, 13]]
9	−1	641,368	C3	[512, 512, 1, False]
10	−1	44,394	Conv	[512, 256, 1, 1]
11	−1	0	Upsample	[None, 2, ‘nearest’]
12	[−1, 6]	0	Concat	[1]
13	−1	77,472	C3	[512, 256, 1, False]
14	−1	5760	Conv	[256, 128, 1, 1]
15	−1	0	Upsample	[None, 2, ‘nearest’]
16	[−1, 4]	0	Concat	[1]
17	−1	16,037	C3	[256, 128, 1, False]
18	−1	52,546	Conv	[128, 128, 3, 2]
19	[−1, 14]	0	Concat	[1]
20	−1	75,473	C3	[256, 256, 1, False]
21	−1	302,680	Conv	[256, 256, 3, 2]
22	[−1, 10]	0	Concat	[1]
23	−1	426,357	C3	[512, 512, 1, False]

**Table 7:** Comparison of YOLOv5-AC and other models’ performances.

Model	AP (%)	Recall (%)	Parameters/10^6^	FLOPS (G)	FPS (f/s)	Model Size/M
Faster R-CNN	71.56	68.40	10.12	20.03	7.1	340 MB
YOLOv4	88.02	86.48	64.45	40.57	39.2	246 MB
YOLOv5s	91.36	90.30	7.06	16.4	40.0	27.2 MB
YOLOv5-AC	95.14	94.22	3.37	9.7	63.1	13.1 MB
Efficientdet-B3	90.48	89.37	13.12	22.3	60.2	68 MB

**Table 8:** Ablation study of YOLOv5-AC with and without channel pruning.

Channel Pruning	P (%)	R (%)	AP (%)	FPS (f/s)	FLOPS (G)	Parameters/106	Model Size (M)
√	97.30	94.87	94.93	63.1	9.8	3.37	13.1
×	97.15	95.07	94.72	40	16.3	7.07	27.6

**Table 9:** Ablation study of YOLOv5-AC with and without AC_FPN.

AC_FPN	P (%)	R (%)	AP (%)	F1 (%)	FLOPS (G)	Parameters/106
√	98.14	94.22	95.12	94.08	9.7	3.37
×	94.25	94.01	91.03	90.15	9.7	3.36

**Table 10:** Ablation study of YOLOv5-AC with and without DIoU_NMS.

DIoU_NMS	P (%)	R (%)	AP (%)	F1 (%)	FLOPS (G)	Parameters/106
√	97.88	94.14	95.03	93.79	9.7	3.37
×	98.01	89.97	95.12	90.24	9.7	3.35

## Data Availability

Not applicable.
